# Enhanced Thermal Pad Composites Using Densely Aligned MgO Nanowires

**DOI:** 10.3390/ma16145102

**Published:** 2023-07-20

**Authors:** Kiho Song, Junhyeok Choi, Donghwi Cho, In-Hwan Lee, Changui Ahn

**Affiliations:** 1 Engineering Ceramic Center, Korea Institute of Ceramic Engineering & Technology (KICET), Icheon 17303, Republic of Korea; 2 Department of Materials Science and Engineering, Korea University, Seoul 02841, Republic of Korea; 3 Advanced Materials Division, Korea Research Institute of Chemical Technology (KRICT), Daejeon 34114, Republic of Korea

**Keywords:** thermal pad composite, thermal conductivity, aligned MgO nanowire, thermal application

## Abstract

Owing to the increasing demand for the miniaturization and integration of electronic devices, thermal interface materials (TIMs) are crucial components for removing heat and improving the lifetime and safety of electronic devices. Among these, thermal pads are reusable alternatives to thermal paste-type TIMs; however, conventional thermal pads comprise a homogeneous polymer with low thermal conductivity. Composite materials of thermally conducting fillers and polymer matrices are considered suitable alternatives to high-performance pad materials owing to their controllable thermal properties. However, they degrade the thermal performance of the filler materials at high loading ratios via aggregation. In this study, we propose novel nanocomposites using densely aligned MgO nanowire fillers and polydimethylsiloxane (PDMS) matrices. The developed nanocomposites ensured the enhanced thermal conducting properties, while maintaining mechanical flexibility. The three-step preparation process involves the (i) fabrication of the MgO structure using a freeze dryer; (ii) compression of the MgO structure; and (iii) the infiltration of PDMS in the structure. The resulting aligned composites exhibited a superior thermal conductivity (approximately 1.18 W m^−1^K^−1^) to that of pure PDMS and composites with the same filler ratios of randomly distributed MgO fillers. Additionally, the MgO/PDMS composites exhibited adequate electrical insulating properties, with a room-temperature resistivity of 7.92 × 10^15^ Ω∙cm.

## 1. Introduction

With the continuous increase in the performance and miniaturization of advanced electronic devices, generated heat has become a major problem that degrades the performance and reduces the lifetime of devices [1]. Thermal interface materials (TIMs) are crucial components that transfer heat from a heat source to a heat sink, ultimately improving the lifespan and safety of electronic devices. Particularly, next-generation optoelectronic devices, such as foldable, flexible, and stretchable devices, require new types of TIMs with adequate flexibility and heat dissipation characteristics [2]. The two main types of TIMs comprise a thermal paste and thermal pad [3,4]. Thermal paste exhibits fluidic properties that allow it to effectively fill the gap between the heat source and heat sink, thereby improving the interfacial heat transfer performance. However, thermal paste moves out of the interface owing to the “pump-out” effect caused by the heat expansion of the interface during device operation, thereby reducing the heat transfer performance and, consequently, degrading the device performance [5,6]. Thermal pads have adequate flexibility and adhesive strength, ensuring their reusability; however, their heat transfer performance is relatively low [7].

A conventional thermal pad comprising a homogeneous polymer has a low thermal conductivity of approximately 0.2 W m^−1^K^−1^. Composite materials composed of fillers and flexible polymeric matrices with high thermal conductivity are suitable alternatives in realizing high-performance thermal pads [8,9]. Thermally conductive filler materials, such as SiO_2_ (1.3 W m^−1^K^−1^) [10,11], Al_2_O_3_ (30 W m^−1^K^−1^) [12,13], AlN (320 W m^−1^K^−1^) [14,15], BN (740 W m^−1^K^−1^) [16,17,18], graphene (2000 W m^−1^K^−1^) [19,20], and carbon nanotubes (CNTs, 3000 W m^−1^K^−1^) [21], have been investigated for improving the thermal conductivity of TIM composites. Although oxide filler materials can be produced at a low cost, they exhibit relatively low thermal conductivity. In particular, nitride fillers have excellent electrical insulation; however, they involve a complex and expensive production process. Graphene and CNTs have excellent thermal conductivity but high electrical conducting properties, which may result in electrical short circuits. Additionally, most composite materials based on particle-type fillers with various morphologies, such as nanoparticles, rods, and nanowires, are manufactured through simple mixing methods for dispersing fillers within polymer matrices. However, realizing efficient and continuous heat transfer pathways using conventional fabrication processes is difficult, owing to the aggregation problems of fillers. Consequently, the thermal conducting performance is degraded at high loading ratios of the filler materials [22,23]. Although the three-dimensional (3D) structuring techniques of fillers have attracted significant attention for maximizing the thermal conducting properties of composites at high filler contents, this approach has certain limitations, including low scalability and high manufacturing costs [24,25,26].

In this paper, we propose a novel high-performance flexible thermal pad fabricated using densely aligned 3D MgO structures. The 3D aligned Mg(OH)_2_ nanowire fillers with moderate thermal conductivity (40 W m^−1^K^−1^) were produced using freeze-casting and uniformly densified via a facile compression process without aggregation. Then, the 3D structures were heat-treated for the transformation of Mg(OH)_2_ to MgO. Subsequently, the surfaces of the densely aligned MgO structures were modified using a silane coupling agent, namely (3-aminopropyl) triethoxysilane (APTES). Finally, polydimethylsiloxane (PDMS) was infiltrated into the remaining pores of the 3D MgO structures. The fabricated thermal pad composites contained a uniformly dispersed and connected MgO filler structure with a filler ratio of up to approximately 11.68 vol%. This resulted in a superior thermal conductivity of approximately 1.18 W m^−1^K^−1^ compared to that of composites with randomly distributed MgO fillers under the same filler ratio. Additionally, the thermal pad exhibited adequate electrical insulation properties (7.92 × 10^15^ Ω∙cm), and demonstrated an outstanding and reliable heat-dissipating performance during the operation of actual devices. Thus, the developed thermal pads have significant potential for thermal management applications in next-generation flexible devices.

## 2. Experimental Section

### 2.1. Fabrication of Mg(OH)_2_ Nanowires

Magnesium acetate tetrahydrate (43.8 g) and urea (5.4 g) were dissolved in 112.5 mL and 337.5 mL of distilled water, respectively, under mechanical stirring at room temperature. Subsequently, the urea solution was added dropwise to the magnesium acetate solution under stirring for 1 h. The mixture was then transferred into a Teflon-lined autoclave (500 mL stainless steel) and heated to 180 °C for 24 h. The product was washed with isopropanol and distilled water using a polytetrafluoroethylene membrane (0.4 μm) and vacuum filtration. Furthermore, the white powder composed of magnesium hydroxide (Mg(OH)_2_) was dried in an oven at 80 °C for 12 h. Finally, the white powder (CH_3_COOH) was debinded in the electrode oven to 310 °C at a heating rate of 3 °C/min for 2 h.

### 2.2. Fabrication of Densely Aligned MgO Structures

Mg(OH)_2_ nanowires (1 g) were dissolved in distilled water (8.8 g) and the dispersant (0.1 g, DISPERBYK-190), followed by the addition of polyvinyl alcohol powder (0.1 g) for 24 h via mechanical stirring at room temperature. The solution was poured into a Teflon mold, and an in-house developed freeze-casting process was used for the experiment. Liquid nitrogen (−196 °C) was used to freeze the copper rod (−60 °C). The Teflon mold was firmly attached to the copper rod, and the samples on the copper rod surface were frozen. After freeze-drying the solution (ilShin Biobase Co., Ltd., Dongducheon-si, Republic of Korea) for 24 h, the Mg(OH)_2_ structure was compressed at a ratio of two, three, and four using a press to obtain different compression conditions. Finally, the product in the electrode oven was calcinated at 450 °C (heating rate of 3 °C/min) for 2.5 h to change the phase from Mg(OH)_2_ to MgO. Then, the 3D structures were sintered at 1400 °C (heating rate of 5 °C/min) for 4 h.

### 2.3. Modification of MgO Structures

We dissolved 1 mL of APTES in 9 mL of distilled water at room temperature under mechanical stirring for 24 h. Subsequently, 90 mL of ethanol was added to the hydrolyzed APTES solution and stirred for 1 h. The MgO structure was modified using 0.05 mol hydrolyzed APTES/EtOH solution at 80 °C for 12 h. Finally, the modified MgO structure was washed several times with methanol and distilled water.

### 2.4. Fabrication of MgO/PDMS Composites

A mixture of PDMS, curing agent, and toluene was prepared at room temperature with a ratio of 10:1:1, respectively. The structure was placed on top of the PDMS mixture, which infiltrated the structure by removing the air within it using a vacuum desiccator for at least 8 h. Finally, PDMS was cured for 5 h at 80 °C. Apart from the random MgO/PDMS composites, other random composites were fabricated for comparison. These composites were composed of 4, 8, and 12 vol% of modified MgO nanowires with PDMS. An identical curing process was followed for the fabrication.

### 2.5. Characterizations

The thermal diffusivity at room temperature was measured using the laser flash method (C, NETZSCH). The morphologies of the 3D MgO structure and composites were measured using a field-emission transmission electron microscope (JSM-7610F, JEOL, Tokyo, Japan). The dielectric constant, loss, and resistivity of the 3D MgO structure were measured using an impedance analyzer and a Faraday cage fixture (Keysight, Santa Rosa, CA, USA, E4990A, 16008B) at various frequencies. A Keysight 16451B dielectric test fixture was used to analyze the change in the dielectric constant with the strain value. The crystal structures of the products were analyzed by examining the MgO nanowire using powder X-ray diffraction (Rigaku D/max-2500, Tokyo, Japan). Fourier-transform infrared spectroscopy (FT-IR) was performed in the range of 500 to 4000 cm^−1^ using a Nicolet 6700 spectrometer. An infrared thermal camera (FLIR E60) was used to measure the change in the temperature of the sample surface over different heating and cooling periods. The mechanical performance of the composites was evaluated with respect to the stress–strain properties. The compressive strength tests of pure the PDMS, random composites, and aligned composites were carried out according to the ASTM D-575-91 standard [27] (sample size: 5 mm × 5 mm × 14 mm) on a universal testing machine (Microload System). Five samples were tested for each composite to collect data on their stress–strain properties under a maximum load of 196.14 N.

## 3. Results and Discussion

**Figure 1:**
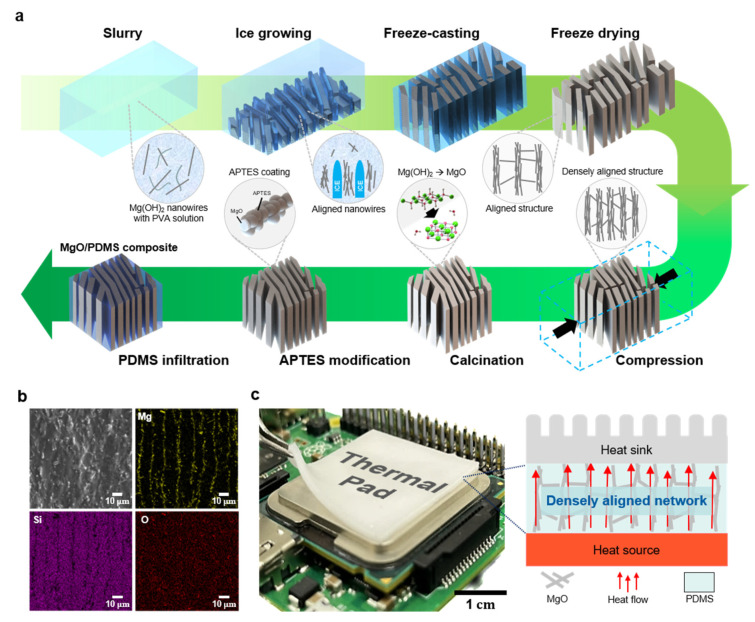
(**a**) Schematic of the fabrication of densely aligned MgO structure-based polydimethylsiloxane (PDMS) composites; (**b**) Energy-dispersive X-ray spectroscopy mapping image of the aligned MgO/PDMS composite; (**c**) Optical image of the thermal pad applied to a central processing unit circuit and schematic of the heat flow in the thermal pad with densely aligned MgO nanowires.

Figure 1a illustrates a schematic of the fabrication process of the densely aligned 3D MgO-based composites. The Mg(OH)_2_ nanowires fabricated via hydrothermal synthesis have a length, thickness, specific surface area, and aspect ratio of 90 μm, 1.5 μm, 19.23 m^2^ g^−1^, and 60, respectively (Figure S1). When MgO was used as the starting material, hydrolysis occurred owing to the moisture exposure during the freeze-casting process and heat treatment (Figure S2). Initially, the Mg(OH)_2_ slurry, where the nanowires and binder were dispersed in water, was used without the phase transition to MgO in order to prevent the hydrolysis of the fabricated 3D structures. As the water in the aqueous solution was frozen through freeze-casting at −60 °C, the fillers were aligned along the ice path. The ice was then sublimated through the freeze-drying process to form the aligned 3D Mg(OH)_2_ structure. The aligned Mg(OH)_2_ structures were evenly densified using a simple uniaxial compression process. Subsequently, the densely aligned Mg(OH)_2_ structures were heat-treated to transform Mg(OH)_2_ to MgO and improve the bonding strength between the fillers. Additionally, the surface of the densely aligned structure was coated with APTES to generate a hydrophobic amino group that can reduce the void in the interface between the MgO filler and PDMS matrix. Finally, the 3D MgO/PDMS composites were realized by introducing PDMS into the structures. After fabricating the composite by infiltrating PDMS into the 3D MgO structure, the cross-section image was examined using energy-dispersive X-ray spectroscopy (EDS) mapping (Figure 1b). The analysis verified the successful penetration of PDMS into the composite without any significant collapse of the MgO structures. The well-aligned MgO fillers promoted the thermal conductivity properties. This structuring technique is a highly efficient strategy to ensure the excellent dispersion and connection properties of the fillers in composite materials. As depicted in Figure 1c, the MgO/PDMS composite was validated by attaching it to the surface of a Raspberry Pi module. The MgO/PDMS composite fabricated using the simple freeze-casting and compression processes exhibited outstanding heat transfer efficiency from the heat source to the heat sink without aggregation at high filler loading ratios.

**Figure 2:**
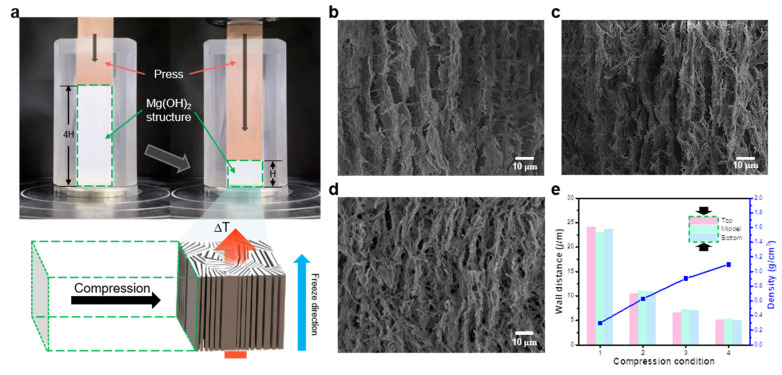
(**a**) Optical image and schematic of the compression process used for fabricating the densely aligned Mg(OH)_2_ structure. Scanning electron microscope images of the MgO structures at compression ratios of (**b**) two, (**c**) three, and (**d**) four. (**e**) Graphs of the density and wall distance at different compression conditions.

To increase the filler content of the 3D structure while preserving the alignment and connectivity of the composite, a facile compression process was applied. The aligned Mg(OH)_2_ structure was uniaxially compressed perpendicular to the freezing direction using a hand press (Figure 2a). The densely aligned MgO structure was fabricated via heat treatment after compression at ratios of two, three, and four, with respect to the initial height of the Mg(OH)_2_ structure. Heat treatment was performed at 1400 °C without a significant collapse of the MgO structures (Figures S3 and S4). As indicated in Figure 2b–d, the density of the 3D MgO structures increases after compression at ratios of two, three, and four; the 3D structures are maintained as the compression ratio is increased to four. Compression at a higher ratio of five (filler ratio of 14.6 vol%) significantly reduced the interwall distance in the MgO structure, hindering the effective penetration of PDMS into the pores of the 3D structure. As a result, the optimal compression ratio was determined to be four. To accurately analyze the uniformity of the degree of densification, the wall distance between the aligned fillers was measured at three regions, namely the top, middle, and bottom of the Mg(OH)_2_ structures (Figure 2e). After the structures were compressed at a ratio of four, the wall distances in the top, middle, and bottom regions were reduced to 78.75%, 76.96%, and 79.23%, respectively. The compressed structures experience a slightly lower stress in the middle region compared with that in the top and bottom regions. Therefore, the compression ratio of the middle region was slightly lower than that of the top and bottom regions. Nevertheless, the structure was uniformly densified with an overall deviation of 1–2%. Furthermore, the density of the fabricated samples increased to 0.3, 0.63, 0.91, and 1.1 g/cm^3^ with an increase in the compression ratios of one, two, three, and four, respectively, further confirming the uniform compression. Thus, a uniformly aligned structure with a high content ratio of approximately 12 vol% was manufactured over an entire area using a facile compression process (Table S1).

**Figure 3:**
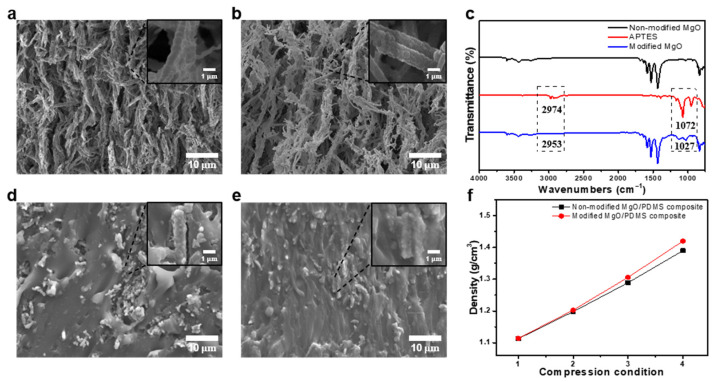
Scanning electron microscope (SEM) images of the (**a**) unmodified and (**b**) modified MgO structures (inset: magnified SEM images); (**c**) Fourier-transform infrared spectroscopy spectra obtained from the unmodified MgO structure, modified MgO structure, and (3-aminopropyl)triethoxysilane (APTES). SEM images of the (**d**) unmodified and (**e**) modified MgO/polydimethylsiloxane (PDMS) composite (inset: magnified SEM images); (**f**) Density of the unmodified and modified MgO/PDMS composites at different compression ratios of two, three, and four.

The surface of the heat-treated MgO structures exhibited a hydrophilic property. Submicron pores were generated in the interface between the MgO fillers and hydrophobic PDMS matrices during the infiltration of PDMS in the 3D MgO structures. The phonon scattering caused by the remaining pores hindered the enhancement effect of the thermal properties. Therefore, to enhance the bonding strength between PDMS and MgO, the hydrophilic surface of the MgO structures was modified to a hydrophobic surface using APTES, which is a silane coupling agent. This modification prevented the hydrolysis of MgO and suppressed the formation of pores at the interface between the fillers and polymer matrices [28]. A thin layer was uniformly coated on the surface of the modified MgO structures without a significant collapse in the structure (Figure 3a,b). Figure 3c illustrates the FT–IR spectra of the unmodified MgO and APTES and modified MgO structures. In the FT–IR spectra of the modified MgO, the 1027 cm^−1^ band indicates the crosslink bonding of Si–O–Mg, whereas the 2953 cm^−1^ band indicates the combination of APTES and MgO with the CH_2_ symmetrical bonding of APTES. To confirm the importance of surface modification using APTES treatment, we analyzed the cross-sectional scanning electron microscope (SEM) images of the MgO/PDMS composites fabricated by filling the MgO structures with PDMS before and after APTES modification (Figure 3d,e). The unmodified MgO/PDMS composite comprised nanopores or micropores at the interface between MgO and PDMS, whereas the number of pores in the modified MgO/PDMS composite was significantly reduced owing to the adequate binding force between MgO and PDMS. As the compression ratio was increased, the difference in the density of the MgO/PDMS composites increased because of the APTES treatment. In particular, the MgO/PDMS composite compressed at a ratio of four, resulting in a suitable interlayer distance, exhibited the largest density difference of 2.16%, owing to the increased probability of pore formation at the MgO/PDMS interfaces (Figure 3f).

**Figure 4:**
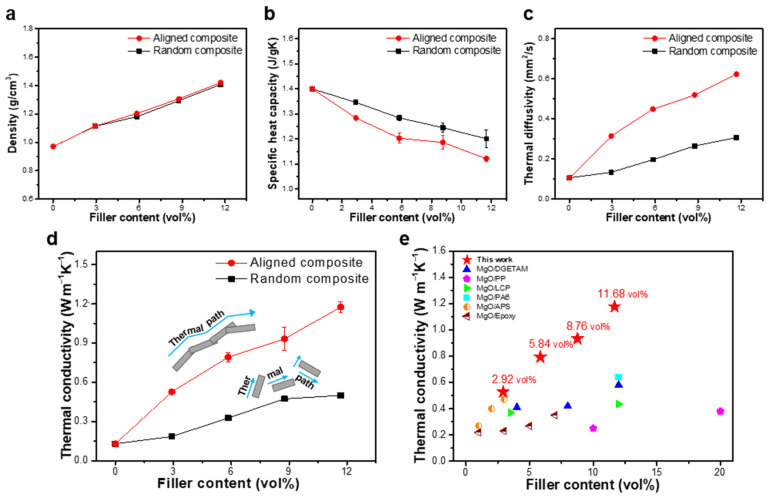
Graphs of the (**a**) density, (**b**) specific heat capacity; (**c**) thermal diffusivity; and (**d**) thermal conductivity of the aligned and randomly distributed MgO/polydimethylsiloxane (PDMS) composites; (**e**) Comparison of the enhancement in the thermal conductivity with respect to previously reported MgO/polymer composites of reference.

The thermal properties of the composites were evaluated by measuring the thermal conductivities of the randomly dispersed MgO composites, 3D densely aligned MgO composites, and pure PDMS. Thermal conductivity (k) can be calculated as follows:

(1) $$k=\rho \;\times \;\alpha \;\times \;C_{P}$$

where ρ, α, and $C_{P}$ denote the density, thermal diffusivity, and specific heat capacity, respectively. Under equal filler ratios, both types of filler-based composites exhibited similar densities owing to the minimization of the pore formation through the APTES treatment (Figure 4a). The specific heat capacity was determined based on the energy consumption of the phonons. The higher the energy consumption is, the higher the specific heat is. Typically, when phonons travel across a material boundary, impurities, and other phonons, they consume energy owing to the scattering effect, resulting in a higher specific heat capacity. In general, the degree of energy consumption of the phonon–boundary migration is substantially higher than those of the phonon–impurity and phonon–phonon migrations. The specific heat capacities of PDMS and MgO are 1.46 and 0.877 kJ kg^−1^K^−1^, respectively. In composites containing randomly dispersed and discrete MgO fillers, phonon travel is primarily affected by the phonon–interface scattering, whereas the phonon travel of the aligned and connected MgO fillers in the composite dominantly occurs via phonon–phonon interactions between MgO fillers. Therefore, the specific heat capacity of the aligned MgO filler-based composites was 7.14% lower than that of the randomly distributed MgO filler-based composite when two types of composites were subjected to the same filler loading ratio (11.68 vol%) (Figure 4b) [29,30]. Additionally, the aligned composites exhibited a thermal diffusivity that is approximately twice that of the random composite, owing to the outstanding connectivity of the MgO fillers (Figure 4c). Therefore, according to the thermal conductivity equation, the thermal conductivity of the aligned composite improved by 136% compared with that of the random composite at the highest filler content (Figure 4d). This structuring approach can effectively improve the thermal characteristics by forming a heat transfer path. We further compared the thermal conductivity of previously reported MgO/polymer composites with that of densely aligned MgO-based composites. Our analysis determined that the aligned composite with 4 vol% filler exhibited a similar or higher thermal conductivity than that of composites with a filler content of 10 vol% or higher. Additionally, the aligned composites with a similar filler content (approximately 12 vol%) were considerably superior (>100%) to previously reported MgO composites (Figure 4e) [31,32,33,34,35,36]. Therefore, the obtained results verified that the proposed technique of manufacturing TIM composites can be an outstanding method for obtaining high-performance thermal pads compared with previously reported MgO polymer composites.

**Figure 5:**
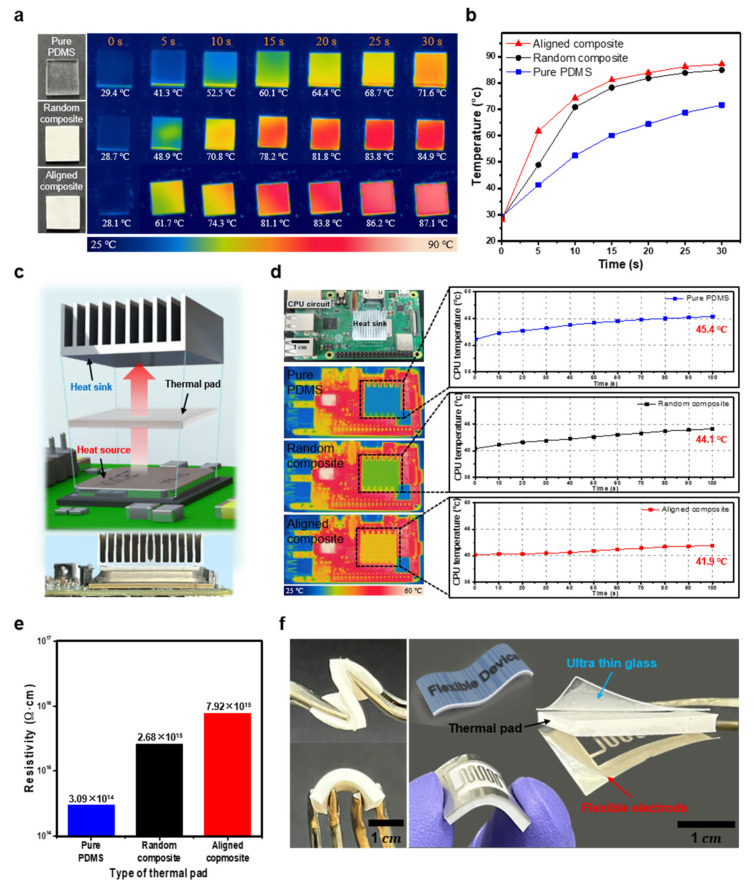
(**a**) Infrared images of the aligned composite (filler ratio of 11.68 vol%), random composite (filler ratio of 11.68 vol%), and pure polydimethylsiloxane (PDMS) during heating; (**b**) Surface temperature curves of the composites and pure PDMS during heating; (**c**) Schematic and optical image of a thermal pad on a central processing unit (CPU) circuit; (**d**) Device performance and Raspberry Pi temperature according to the type of thermal pad; (**e**) Direct current electrical resistance based on the type of thermal pad; (**f**) Optical images indicating the flexibility of the MgO/PDMS composite.

The applicability of the developed composite to actual devices was evaluated by analyzing the heat dissipation, insulation, and mechanical properties of the pure PDMS, random composites, and aligned composites. The heat dissipation performance of the composites was examined by measuring their surface temperature using an infrared thermal imaging camera under constant heating at 90 °C. The pure PDMS, aligned composites, and random composites were placed on a hot plate (90 °C), and the changes in the temperature distribution of the surface were recorded for 30 s using a fixed thermal imaging camera (Figure 5a). The temperature increase in the aligned composite was 1.49 and 1.26 times faster within the initial 5 s than that of the pure PDMS and random composites, respectively, owing to its higher thermal conductivity. After 10 s, the temperature changes significantly decreased for all three samples. After 30 s, the recorded surface temperatures of the aligned and random composites were 87.1 and 84.9 °C, respectively, indicating the improved thermal conducting behavior of the aligned composite (Figure 5b). The difference in the thermal conductivity of the composites is dependent on the filler network within the material, which can be verified by examining the cross-section images of the composite (Figure S5).

Figure 5c illustrates the schematic and optical image of the heat flow, wherein the heat from the heat source of the central processing unit (CPU) circuit passes through the thermal pad and flows into the heat sink. To demonstrate the heat-dissipating performance of the composite materials in practical devices, the composites and pure PDMS were inserted between the CPU and heat sink. The temperatures of the heat sink and CPU were measured during the device operation using an internal CPU temperature measurement program via a thermal-imaging camera. The temperature of the heat sink with the aligned composite pad was higher than that with the pure PDMS and random composite, owing to the adequate heat transfer efficiency of the aligned composite. After 100 s of device operation, the temperature of the internal chip with the aligned pad decreased by 3.5 and 2.2 °C compared to that of the internal chip with pure PDMS and the random composite, respectively (Figure 5d). Therefore, the aligned composite can be considered a superior heat-dissipating pad over pure PDMS and random composites, facilitating effective heat dissipation within the CPU while maintaining relatively low temperatures.

Another requirement for thermal pads applied to electronic devices is electrical insulation to prevent the electrical hazards caused by the leakage current of electronic devices. The resistivity of the two composites and pure PDMS was measured by applying a direct current. The fabricated 3D composites demonstrated an enhanced compressive strength compared to the random composites and pure PDMS (Figure S6). The aligned MgO composites exhibited high electrical resistivity, which ensured adequate insulating properties (Figure 5e). The thermal conductivity of the composites with different fillers enhanced the thermal conductivity with respect to the filler content, similar to the electrical resistance (Figure S7) [37,38,39]. Thus, the developed composites can be used as thermal pads between flexible electrodes and thin glasses owing to their exceptional adhesive properties and flexibility (Figure 5f), facilitating their application in next-generation flexible and stretchable electronic devices.

## 4. Conclusions

In this study, we developed a thermal pad composite with superior thermal, mechanical, and electrical properties using densely aligned MgO filler structures without aggregation problems. The densely aligned thermal pads enhanced the thermal conductivity by 136% compared with the randomly dispersed thermal pads at the highest filler content (11.68 vol%). The aligned thermal pad composites also demonstrated improved heat dissipation characteristics when applied to practical electronic devices. Therefore, the proposed thermal pad composite can serve as an efficient and excellent thermal management material for next-generation devices, such as curved, flexible, and stretchable devices, owing to its exceptional electrical insulation, flexibility, and adhesion. Furthermore, unique and outstanding composites produced through the 3D structuring of different filler materials can be applied to various applications, such as electromagnetic shielding materials (copper), transparent conductive flexible substrates (indium tin oxide), and high-strength structural materials (carbon fiber).

## Data Availability

The data presented in this study are available in supplementary material here.

## Supplementary Materials

The following supporting information can be downloaded at: https://www.mdpi.com/article/10.3390/ma16145102/s1, Figure S1. Elemental analysis of precursor Mg(OH)_2_ nanowires by hydrothermal synthesis: (a) optical image, (b) SEM image with low magnification, and (c) X-ray diffraction pattern. Figure S2. (a) SEM image and (b) X-ray diffraction pattern of hydrolyzed MgO structure. Figure S3. (a) An SEM image of the Mg(OH)_2_ structure, and (b) graphs of density and wall distance according to calcination temperature. The Mg(OH)_2_ structures with heat treatment temperatures of (c) 1200 °C, (d) 1300 °C, (e) 1400 °C, and (f) 1500 °C. Figure S4. The X-ray diffraction patterns of (a) before calcination and (b) after calcination to MgO. Figure S5. Cross-sectional SEM images and schematics of the (a,d) Pure PDMS, (b,e) random composite, (c,f) aligned composite. Figure S6. Stress-strain curves for three-type of thermal pad materials. Figure S7. (a) Comparison of the thermal conductivity and (b) electrical insulation properties with previously reported materials composites. Table S1. The graphs of density and wall distance according to compression conditions.
