# Supplementary material for: Enhanced Thermal Pad Composites Using Densely Aligned MgO Nanowires
Source: Materials (Basel). 2023 Jul 20;16(14):5102. doi: 10.3390/ma16145102 (PMC10386388; doi:10.3390/ma16145102)
Supplement: Supplementary file 1 [file materials-16-05102-s001.zip › materials-2506747-supplementary.pdf]

Supplementary Information

Enhanced Thermal Pad Composites Using Densely Aligned MgO Nanowires

Kiho Song [1,2], Junhyeok Choi [1], Donghwi Cho [3], In-Hwan Lee [2,*] and Changui Ahn [1,*]

1 Engineering Ceramic Center, Korea Institute of Ceramic Engineering & Technology (KICET), Incheon 17303, Republic of Korea

2 Department of Materials Science and Engineering, Korea University, Seoul 02841, Republic of Korea

3 Advanced Materials Division, Korea Research Institute of Chemical Technology (KRICT), Daejeon 34114, Republic of Korea

* Correspondence: ihlee@korea.ac.kr (I.-H.L.); acu2001@kicet.re.kr (C.A.)

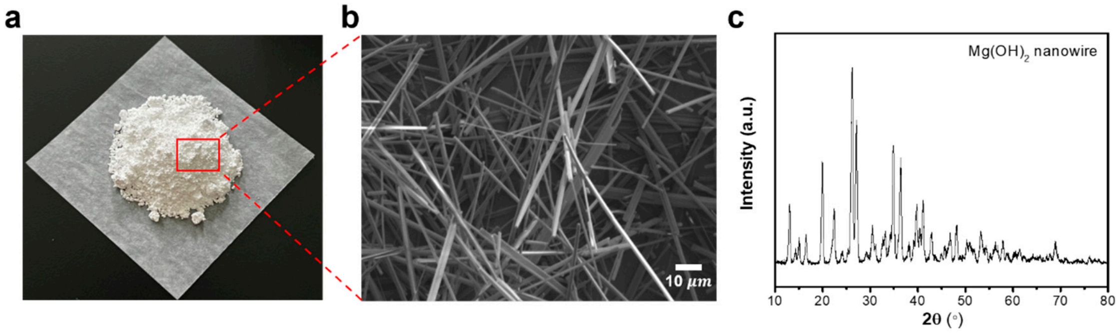

Fig. S1. Elemental analysis of precursor $Mg(OH)_2$ nanowires by hydrothermal synthesis: (a) optical image, (b) SEM image with low magnification, and (c) X-ray diffraction pattern.

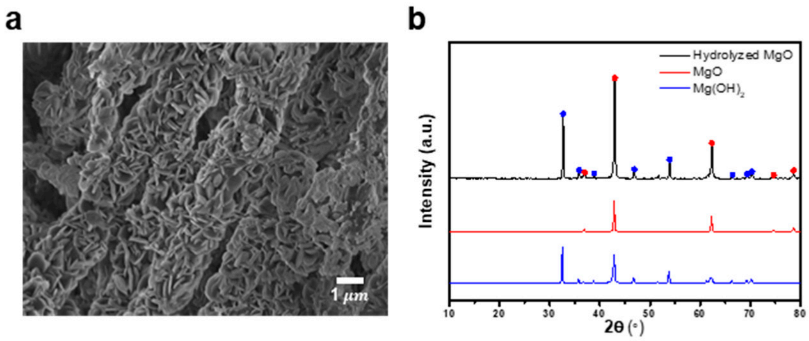

Fig. S2. (a) SEM image and (b) X-ray diffraction pattern of hydrolyzed MgO structure.

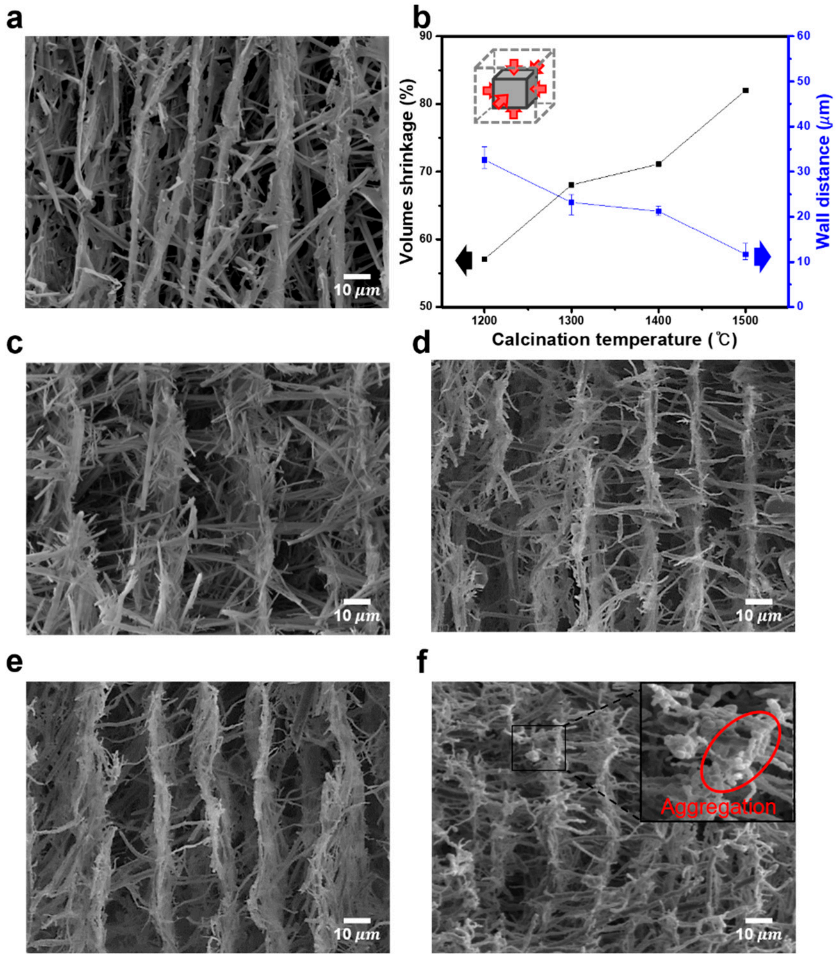

Fig. S3. (a) An SEM image of the $Mg(OH)_2$ structure, and (b) graphs of density and wall distance according to calcination temperature. The $Mg(OH)_2$ structures with heat treatment temperatures of (c) 1200 °C, (d) 1300 °C, (e) 1400 °C, and (f) 1500 °C.

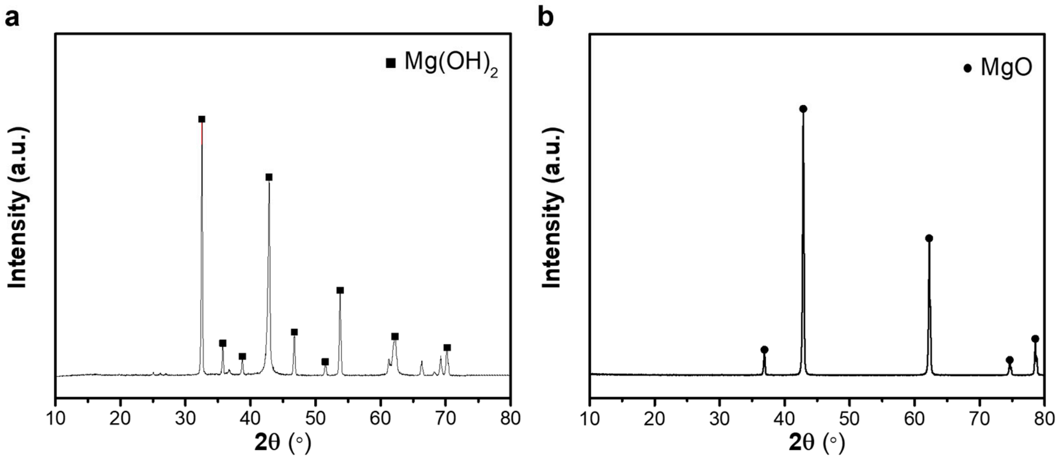

Fig. S4. The X-ray diffraction patterns of (a) before calcination and (b) after calcination to MgO

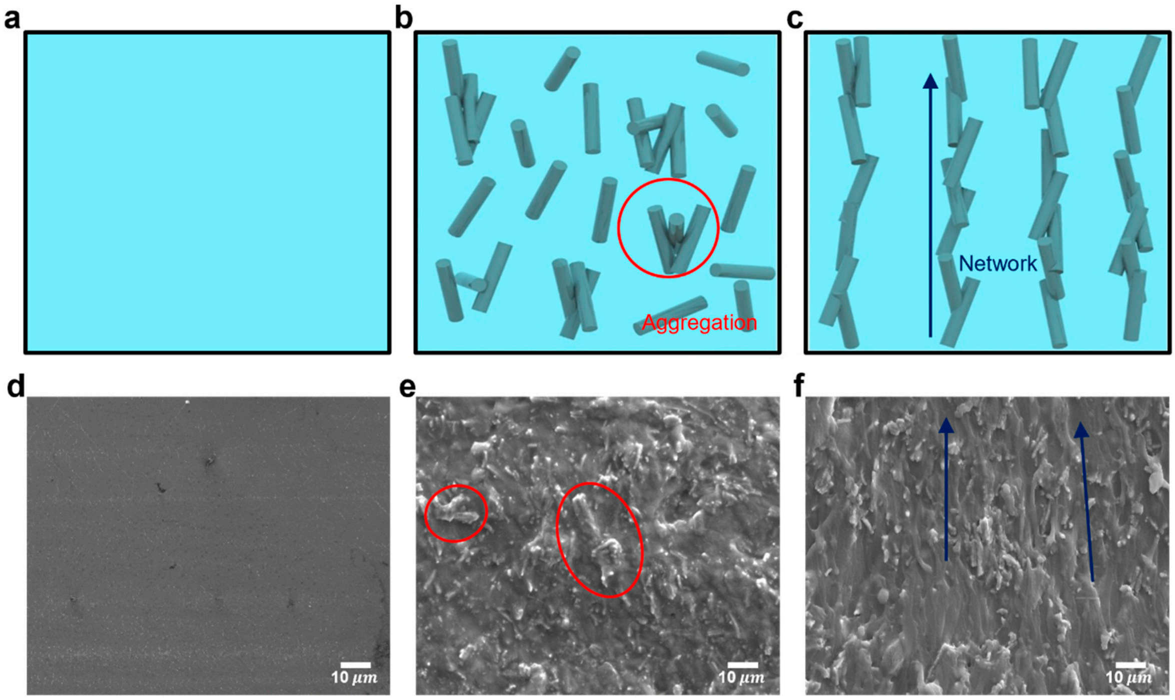

Fig. S5. Cross-sectional SEM images and schematics of the (a, d) Pure PDMS, (b, e) random composite, (c, f) aligned composite.

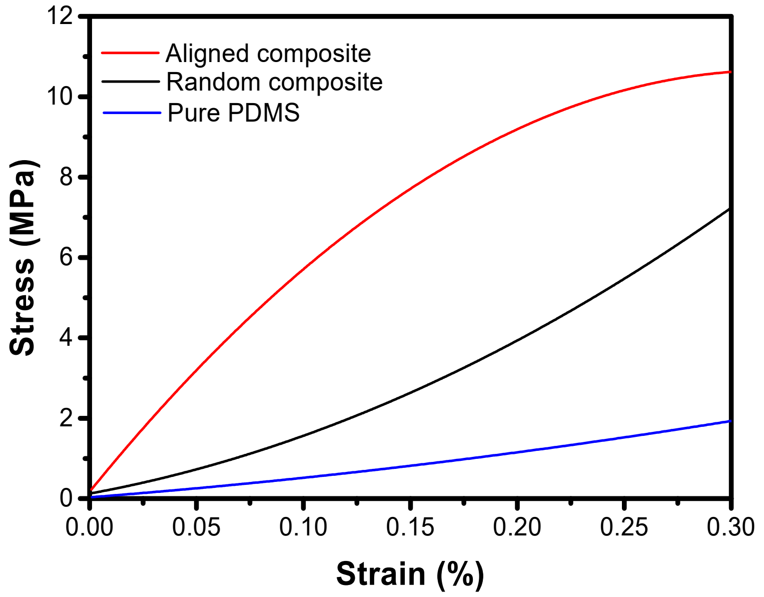

Fig. S6. Stress-strain curves for three-type of thermal pad materials.

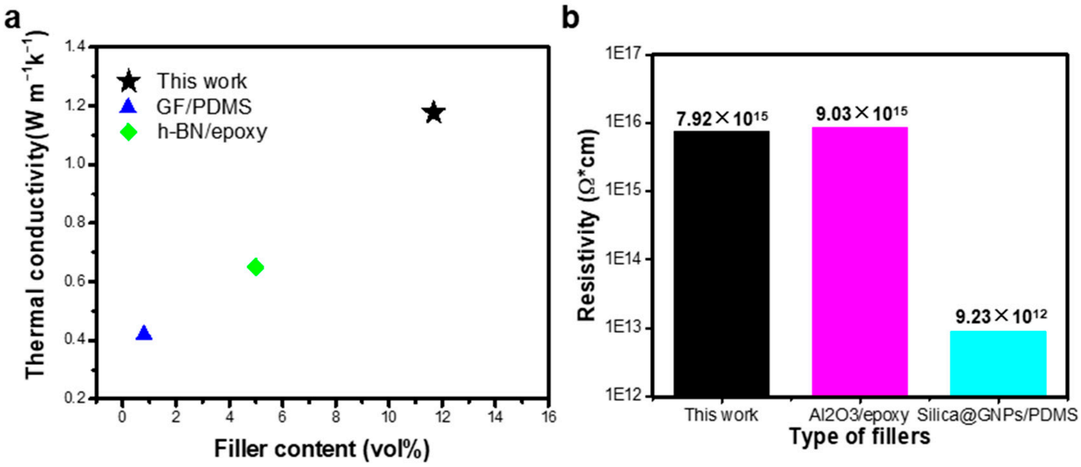

Fig. S7. (a) Comparison of the thermal conductivity and (b) electrical insulation properties with previously reported materials composites.

compression condition	Wall distance (μm)			Density (g/cm^3)
	Top	Middle	Bottom	
×1	24	23	23.6	0.3
×2	10.45	11	10.8	0.63
×3	6.54	7.2	6.96	0.91
×4	5.1	5.3	4.9	1.1

Table S1. The graphs of density and wall distance according to compression conditions.
